# Application of Bioactive Coatings with Killer Yeasts to Control Post-Harvest Apple Decay Caused by *Botrytis cinerea* and *Penicillium italicum*

**DOI:** 10.3390/foods11131868

**Published:** 2022-06-24

**Authors:** Urszula Błaszczyk, Sylwia Wyrzykowska, Maciej Gąstoł

**Affiliations:** 1 Department of Fermentation Technology and Microbiology, Faculty of Food Technology, University of Agriculture in Krakow, Aleja Mickiewicza 21, 31-120 Krakow, Poland; sylwia.wyrzykowska@urk.edu.pl; 2 Department of Horticulture, Faculty of Biotechnology and Horticulture, University of Agriculture in Krakow, Aleja Mickiewicza 21, 31-120 Krakow, Poland; maciej.gastol@urk.edu.pl

**Keywords:** biological control, killer yeast, *Botrytis cinerea*, *Penicillium italicum*, green practices and processes

## Abstract

A new method was proposed to produce alginate bio-films containing *Pichia membranifaciens* and *Wickerhamomyces anomalus* killer yeast to control the post-harvest fungal decay in organic apples caused by *Botrytis cinerea* and *Penicillium italicum*. Coatings with *W. anomalus* killer yeast effectively controlled the growth of *P. italicum* during storage at 22 °C. *W. anomalus* killer yeast incorporated in alginate reduced the *P. italicum* incidence from 90% (control) to 35% after 14 days of storage at 22 °C. Alginate biofilms with *W. anomalus* or *P. membranifaciens* also limited the incidence of the fungal decay of apples inoculated with *B. cinerea* compared with the control fruits, although the antagonistic capability against *B. cinerea* was lower than against *P. italicum*. The survival of *W. anomalus* cells in alginate coating was higher than *P. membranifaciens*. The incorporation of killer yeasts into alginate had no significant effect on the mechanical properties (tensile strength, percent elongation at break) of alginate coating, however, they increased the thickness of the biofilm. The bioactive coating reduced the fruit weight loss and had no significant effects on the fruit firmness during storage at 2 °C. As organic apples, produced without any synthetic fungicides, are especially prone to fungal decay during storage, the proposed alginate biofilms containing killer yeast seem to be a very promising solution by offering non-chemical, biological control of post-harvest pathogens.

## 1. Introduction

Fruits and vegetables during their growth and storage are exposed to diseases caused by fungal pathogens. The consequence of the occurrence of mycoses is a reduction in the crop and deterioration of their quality. During storage, the losses of apple fruits caused by fungal pathogens are estimated to be 5 to 25% of the initial quantity [1]. In addition to the economic losses, the growth of mold in food reduces its nutritional value, moreover, it can become contaminated with dangerous mycotoxins [2]. To prevent fungal diseases, fungicides are widely used, the advantages of which are high efficiency and ease of application. However, the chemical control of post-harvest diseases by fungicides can have many negative effects and increasing concerns are arising about their application. Residues of fungicides in plants can negatively affect the health and safety of consumers. The use of fungicides causes environmental pollution and may lead to the increase in the resistance of phytopathogens to chemical agents and the reduction in beneficial organisms. This is why new solutions and strategies are still being sought after, and biological plant protection methods are of great interest. Biological control is associated with limiting the development of harmful organisms by the application of other organisms or their metabolic products. The biocontrol of plant diseases of fungal origin may include the use of antagonistic yeasts that can be considered as one of the potential safe alternatives to chemical fungicides [2]. Biocontrol yeasts demonstrate antagonistic action against fungal pathogens through several mechanisms such as competition for limiting nutrients (e.g., carbohydrates, nitrogen, iron cations) and space, the production of antifungal metabolites (diffusible and volatile compounds), the release of lytic enzymes (e.g., chitinases and glucanases) and mycoparasitism, the induction of host resistance, biofilm formation, and most recently described, the involvement of oxidative stress [3,4,5]. The successful inhibition of the pathogen infection and development may be the result of multiple mechanisms occurring simultaneously. The final effect is dependent on the complex interactions between all components of the biological control system such as the plant host, antagonist, pathogen, and resident microflora [5].

Promising results of the antagonistic activity of yeast as a means of natural biological control in laboratory experiments resulted in the registration of *Candida oleophila* in 1995 by the U.S. Environmental Protection Agency as a post-harvest antifungal agent [6], which paved the way for the commercialization of yeast-containing biocontrol products. Among the registered and commercially available first-generation biocontrol products, in addition to *C. oleophila* (Aspire, Ecogen, Langhorne, PA, USA), there are also yeasts such as *Cryptococcus albidus* (YieldPlus, Lallemand, Montreal, QC, Canada) and *Candida sake* (Candifruit, IRTA, Caldes de Montbui Barcelona, Spain) [7]. A preparation containing two antagonistic strains of *Aureobasidium pullulans*, which is a yeast-like fungus (BoniProtect, Konstanz, Germany) has also been developed for pre-harvest use to combat pathogens of developing wounds in apples during storage [5,8].

In the recent two decades, several studies have also reported the incorporation of living yeasts in edible films and coatings primarily to control post-harvest diseases. These films and coatings are not only designed to maintain the yeast population and their antagonistic effect, but also to improve the shelf life and quality of food including creating a protective barrier against physical and mechanical damage and providing a controlled atmosphere and acting as a semi-permeable barrier to gases, vapor, and water [8]. They are produced using natural origin materials such as mainly polysaccharides, but also proteins and lipids or a mixture of these materials. Usually made from byproducts from agricultural and marine sources, to some extent, they can replace food packaging made from a non-renewable source, thus reducing the number of waste products and meeting the environmental requirements [8]. Alginate is one of the film-forming biopolymers, and is a natural polysaccharide extracted from brown algae. The molecular structure of alginate is composed of unbranched, linear copolymers of β-D-mannuronic acid and α-L-guluronic acid residues linked by 1–4 glycosidic bonds [9]. Alginate has good film-forming properties and is an excellent moisture barrier [10]. It can be used to increase the shelf life of fruits and vegetables by reducing dehydration, controlling the respiration process and improving the mechanical properties [9].

Several studies have reported the results on the application of coatings containing antagonist yeast to the surface of the fruit. One example of the incorporation of living yeast into a coating for biocontrol purposes is the use of *C. oleophila* in cellulose (methylcellulose or hydroxypropylcellulose) films [11] or shellac and sucrose ester based coatings [12,13] to extend the storage of grapefruits. To reduce the post-harvest decay of oranges, cellulose-based coatings as carriers for *Candida guillermondii* and *Debaryomyces* sp. were used [14]. In other studies, coatings with locust bean gum and sodium alginate with incorporated *Wickerhamomyces anomalus* [15] as well as coatings based on chitosan and locust bean gum containing pomegranate peel extract locust bean gum and with *W. anomalus* [16] were tested. Satisfactory results of the biocontrol of *Penicillium digitatum* and *P. italicum* on mandarin fruit were obtained after applying coatings based on locust bean gum containing *W. anomalus*, *Metschnikowia pulcherrima*, and *A. pullulans* [17]. For the management of post-harvest diseases of Persian limes, cross-linked arabinoxylans coatings with *Debaryomyces hansenii* entrapped were tested [18]. An alginate coating with *Cryptococcus laurentii* was used to extend the shelf life of the strawberries [19]. There are a few examples of the application of coatings with antagonistic yeast to reduce the post-harvest decay of apples. A recently published article describes the use of apple-based coatings with *M. pulcherrima* yeast isolated from wild apples [20].

The objective of this work was to investigate the potential application of alginate coatings with *Pichia membranifaciens* or *Wickerhamomyces anomalus* killer yeasts to control the post-harvest fungal decay caused by *Botrytis cinerea* and *Penicillium italicum* in organic apples. Additionally, the effect of these bioactive coatings on other post-harvest quality parameters of fruits such as weight loss, firmness, soluble solids content, and titratable acidity was evaluated.

## 2. Materials and Methods

### 2.1. Microorganisms

The killer yeast strain of *Wickerhamomyces anomalus* CBS 1982 was provided from the CBS-KNAW Collections, Westerdijk Fungal Biodiversity Institute (Utrecht, The Netherlands). The killer yeast strain of *Pichia membranifaciens* LW26 was isolated from apple fruits of cultivar Koksa Górska (Łososina, Poland), which was harvested in the middle of October 2016 [21]. Similarly, *Penicillium italicum* LW75 was isolated from diseased apple fruits of the cultivar Szara Reneta (Grójec, Poland), harvested in the middle of October 2016. *Botrytis cinerea* Łock 0463 was obtained from the collection of the Institute of Fermentation Technology and Microbiology, Łodz University of Technology (Poland). The killer sensitive strain *Saccharomyces cerevisiae* DBVPG 6500 was provided by Industrial Yeasts Collection DBVPG (Perugia, Italy).

Yeast cultures were grown on YPD agar slants (1% (*w/v*) yeast extract, 2% (*w/v*) peptone, 2% (*w/v*) glucose, and 2% (*w/v*) agar), and maintained at 4 °C. The fungi were grown in Petri dishes with potato-dextrose agar (PDA) and incubated for seven days at 25 °C.

### 2.2. Fruits

‘Topaz’ apples (Malus domestica Borkh.) were harvested on 20 October 2017 from an organic orchard in Garlica (near Krakow, Poland). The visual selection of fruits was based on the shape uniformity, size (75–80 mm diameter), and color as well as the absence of injury or fungal infection.

### 2.3. Determination of Killer Activity

The killer activity assay was performed according to Stumm et al. [22], with some modifications. The cells of the sensitive yeast strain (*S. cerevisiae* DBVPG 6500) were grown for 24 h at 25 °C on YPD agar slants. Next, the suspension of sensitive yeasts in sterile water was mixed with the molten YPD-MB medium containing 0.003% (*w/v*) methylene blue adjusted to pH 4.5 (the optimal pH for killer toxin activity) with 0.1 M citrate-phosphate buffer. The final cell concentration of the sensitive strain was approximately 2 × 10^5^ cells per mL of the assay medium. A single streak of the tested strains was conducted on the YPD-MB agar plates, which were incubated at 25 °C for 48–72 h. The appearance of a clear zone of growth inhibition around the streak of the tested strain, bounded by bluish-stained cells was recorded as a presence of killer activity. Since the action of some killer toxins depends on the presence of NaCl, the assay was performed with (3%) and without NaCl [23].

### 2.4. In Vitro Antagonistic Activity of Yeasts against Fungi

The antagonistic activity of the *W. anomalus* and *P. membranifaciens* killer strains against *P. italicum* and *B. cinerea* was investigated by the in vitro assay according to Perez et al. [23], with some modifications. In brief, a 5 mm diameter plug of the fungal pathogen, taken from the edge of an actively growing colony, was placed on 20 mL of PDA medium (buffered to pH 4.5) at the center of the Petri dish. Subsequently, a loop of the yeast cells was positioned at adjacent sites, 2 cm from the center of each Petri dish. Plates were incubated at 25 °C for 10 days and the diameters of the fungal growth were periodically measured. The percentage of mycelial growth inhibition by the killer yeast was estimated compared to the control (mycelial growth of fungal pathogen alone), according to the following formula (Equation (1)):

Inhibition (%) = [(dC − dT)/dC] × 100% (1)

where dC (cm) is the mean of the colony diameter for the control and dT (cm) is the mean of the colony diameter in the presence of the antagonistic yeast. The experiment was performed with six replicates.

### 2.5. In Vitro Antifungal Activity of Yeast Volatile Organic Compounds (VOCs)

The antagonistic activity of VOCs produced by the *W. anomalus* and *P. membranifaciens* killer strains against the tested pathogenic fungi was determined by the dual culture method, in which the fungus and the biocontrol yeasts were not in physical contact. The assay was performed according to the method described by Nally et al. [4], with some modifications. Petri plates containing 20 mL of solid YPD medium were seeded with each killer yeast strain. At the same time, mycelial plugs of *P. italicum* or *B. cinerea* (5 mm in diameter), taken from the edge of an actively growing colony, were placed in the center of the PDA plates. Next, the covers of both of the inoculated dishes were removed and the sets of double plates were sealed using Parafilm to obtain a double-dish chamber. As a control, the sets with the closed double plates without the killer yeast cultures were also prepared. The plates were incubated at 25 °C for 5 days, after which the fungal growth diameter was measured. The percentage of fungal inhibition was estimated compared to the control without the killer yeast according to Equation (1). These experiments were performed in eight replicates.

### 2.6. Preparation of the Bioactive Films

The film-forming solutions were prepared using sodium alginate (CAS No 9005-38-3, Sigma Aldrich: Steinheim, Germany; 2% *w/v*) as a structural polymer of the film and glycerol (0.24 g/g of alginate) as a plasticizer, reducing the brittleness and improving the film flexibility. Initially, the coating solution (2% sodium alginate) was heated at 70 °C with constant agitation until all of the powder was completely dissolved. Subsequently, glycerol was added to the solution and the mixture was left to stir for 1 h at room temperature. Then, the cells of selected killer yeasts were added to a film-forming solution. *W. anomalus* or *P. membranifaciens* killer yeasts were used for the preparation of bioactive films. Yeasts were cultivated in YPD liquid medium overnight and cells were harvested by centrifugation at 2500× *g* for 20 min and washed with sterile water. Subsequently, the killer strain was incorporated by adding the suspension of yeast cells into the alginate coating solution and the mixture was stirred for 10 min. The concentration of the killer yeast cells was fixed to have a final concentration of ~6 logs CFU/cm^2^ in the bioactive film. The film-forming solutions (20 mL) were poured onto Petri dishes (90 mm diameter) and allowed to dry at 30 °C.

### 2.7. Evaluation of Viability of Killer Yeasts during Storage

The viability of *W. anomalus* CBS 1982 and *P. membranifaciens* LW26 killer yeasts incorporated in biofilms were assessed just after the biofilm was dried and every seven days during storage periods of four weeks at 2 ± 0.5 °C, and subsequently 14 days at 22 ± 1 °C. Biofilms were aseptically removed from Petri dishes, cut, and placed into a sterile flask containing sterile saline solution (0.9% NaCl, 20 mL). Next, the solutions were stirred for 6 h at 25 °C to dissolve the biofilm and allow for the cells of thee microorganisms to be released into the saline solution. Subsequently, serial decimal dilutions of the samples were prepared. A 0.1 mL sample of appropriate dilutions was spread on YPD agar with the addition of 0.1 g L^−1^ chloramphenicol. The YPD plates were incubated for 48 h at 25 °C, and then colonies of yeasts were counted. The assays were performed in triplicate.

### 2.8. Thickness and Mechanical Properties of Alginate Films

The coating thickness was determined using a digital micrometer. The average values were taken from measurements that were made at five different locations of each sample. The tensile strength (TS) and percent elongation at break (%E) were measured with a texture analyzer (TA-XT2 plus, Stable Micro Systems, Godalming, UK) according to a standard test method D882 [24]. The alginate films were cut into 20 mm wide and 100 mm long strips, and mounted between the grips with an initial separation of 50 mm, and the cross-head speed was set to 60 mm/min. The tensile strength (TS) was determined by dividing the maximum load required to break the film sample by the original minimum cross sectional area. The percent elongation at break (%E) was calculated by dividing the extension at the moment of rupture by the initial gauge length of the film sample and multiplying by 100. Eight replicates of each type of film were examined.

### 2.9. Application of Coating Treatments and Control of Fungal Spoilage of Apples

The apples were washed with 0.01% sodium hypochlorite solution for 3 min, then drained and air-dried at room temperature. Subsequently, the fruits were injured (three random wounds per apple) and inoculated with a conidial suspension of 5 × 10^4^ spores mL^−1^ of *P. italicum* or *B. cinerea* (the number of spores was counted using a Thoma chamber). Wounds were about 3 mm deep and 3 mm wide, and were inoculated with 20 μL of the spore suspension. After drying at room temperature for 3 h, the apple fruit was randomly assigned to different treatments, named CP: uncoated fruit, injured, and inoculated with *P. italicum*; CPA: fruit injured and inoculated with *P. italicum*, coated with 2% alginate; PAW: fruit injured and inoculated with *P. italicum*, coated with 2% alginate containing *W. anomalus*; PAP: fruit injured and inoculated with *P. italicum*, coated with 2% alginate containing *P. membranifaciens*; CB: fruit injured and inoculated with *B. cinerea*; CBA: fruit injured and inoculated with *B. cinerea*, coated with 2% alginate; BAW: fruit injured and inoculated with *B. cinerea*, coated with 2% alginate containing *W. anomalus*; BAP: fruit injured and inoculated with *B. cinerea*, coated with 2% alginate containing *P. membranifaciens*. Three replicates of 10 apples were used for each treatment.

The fruits were dipped in the coating solution for 1 min. After the coating treatments, the apples were hung up and air-dried at room temperature for 24 h. The fruit was stored at 2 ± 0.5 °C and 85–90% relative humidity for four weeks. Subsequently, apples were stored at 22 ± 1 °C and 45% RH for 14 days to simulate retail handling and market conditions. The fungal growth and the disease incidence, expressed as the number of infected apples per total number of fruits in each treatment, was evaluated every two days until the end of the incubation time. If at least one of the three inoculated wounds was infected, the fruit was considered decayed.

### 2.10. Quality Parameters of Apples Uncoated and Coated with Alginate Biofilms

The weight of fruit (fifteen apples for each treatment) was measured in grams using an electronic scale and the results were estimated as the percentage of weight loss. Flesh firmness was measured on opposite sides of the fruits using an Effegi penetrometer (model 327, with 10 mm plunge. The soluble solid content was ascertained in freshly prepared juice using an Atago PR-101 digital refractometer (AOAC 932.12). Titratable acidity (TA) was measured by titrating with 0.1 M NaOH to a pH end-point of 8.1 using a CX-501 multifunction meter (Elmetron) [25], according to the following formula (Equation (2)):

TA (g L^−1^) = N × V_1_ × Eq.wt/V_2_ × 10 (2)

where TA is the titratable acidity; N is the normality of the titrant (mEq/mL); V_1_ is the volume of the titrant (mL); V_2_ is the volume of the sample (mL); and Eq.wt. is the equivalent weight (134) of malic acid. Analyses were performed in triplicate.

### 2.11. Statistical Analysis

The data were subjected to statistical analysis, the mean values and standard deviations were determined, and the significance of the variables was assessed. Statistically significant differences between means (*p* < 0.05) were evaluated using the analysis of variance (ANOVA) with a post hoc Tukey–Kramer’s test. InStat software, version 3.01 (GraphPad Software Inc., San Diego, CA, USA) was applied for statistical analyses of the results.

## 3. Results and Discussion

### 3.1. Killer Activity

The production of killer toxins is one of the mechanisms that is of crucial importance in the biocontrol activity of antagonistic yeasts against other microorganisms. A clear zone of sensitive yeast growth inhibition bounded by bluish-stained cells indicates the presence of killer activity. Strains of *W. anomalus* and *P. membranifaciens* selected for the further stage of the experiments were characterized by the highest killer activity among the tested yeast strains isolated from apples and obtained from the yeast collections. It was estimated that about a quarter of the strains isolated from the fruit showed a killer phenotype [26]. Numerous studies have confirmed that killer yeasts had a great potential as biocontrol agents and can protect fruit in both the pre- and post-harvest stages [27]. Killer yeast has also been used to control plant pathogens causing post-harvest diseases on fruits such as citrus, papaya, grape, stone fruit, and apple [23,27,28,29,30].

### 3.2. In Vitro Efficacy of Yeast Strains against Decay-Causing Fungi

Antagonistic yeasts can produce various diffusible and volatile compounds with inhibitory activity against fungal pathogens [5]. The results presented in Table 1 demonstrate that two tested killer yeasts produce agar-diffusible antifungal metabolites. Both *W. anomalus* and *P. memebranifaciens* strains significantly reduced the mycelial growth of *P. italicum* and *B. cinerea*. After 10 days of incubation, *W. anomalus* and *P. memebranifaciens* decreased the growth of *P. italicum* by 56% and 38%, respectively. The killer yeasts also limited the development of *B. cinerea* with 53% and 64% growth inhibition by *W. anomalus* and *P. memebranifaciens,* respectively. The results demonstrated that *W. anomalus* had higher antagonistic activity against *P. italicum*, while *P. memebranifaciens* was against *B. cinerea*.

Previously, other reports have shown that *W. anomalus* exhibited antifungal activity against *Penicillium roqueforti* during the storage of cereal grains [31,32] and reduced the ochratoxin A accumulation in co-culture with *Penicillium verrucosum* [33]. It was also found that *W. anomalus* and *Lactobacillus plantarum* in a mixed starter culture during sourdough fermentation reduced the fungal contamination of wheat flour bread until 28 days of storage at room temperature [2]. In the case of *P. membranifaciens*, some research indicated that yeasts from the mentioned species can inhibit *B. cinerea* growth [34,35].

### 3.3. Antifungal Activity of Yeast Volatile Organic Compounds

The results of double plate assays demonstrated that the tested killer yeasts can produce volatile compounds with antifungal activity. The volatiles from the killer yeast was effective in reducing the mycelial growth of *P. italicum* and *B. cinerea* (Table 1). After incubation for 5 days, due to the presence of the volatiles from the cultures of *W. anomalus*, the average colony diameter of *P. italicum* and *B. cinerea* was reduced by 51% and 70%, respectively. The volatile compounds produced by *P. membranifaciens* inhibited the growth of *P. italicum* and *B. cinerea* by 21% and 40%, respectively.

The synthesis of volatile organic compounds (VOCs) can act in an important role in inhibiting mold growth by *W. anomalus* [36]. Oro et al. [37] demonstrated that volatiles produced by *W. anomalus* inhibited mycelial growth of *B. cinerea* by 87% and also reduced the development of *Monilinia fructicola*, *Alternaria alternata*, *Aspergillus carbonarius*, *Penicillium digitatum*, *Cladosporium* spp., and *Colletotrichum* spp. In particular, ethyl acetate is considered as one of the key inhibitory compounds synthesized by *W. anomalus*. This compound released by *W. anomalus* has been shown to have antifungal activity during the airtight storage of grain [38]. The vapors of ethyl acetate completely stopped the growth of *B. cinerea* at 8.97 mg mL^−1^ and reduced grey mold incidence on strawberry fruits at 0.718 mg mL^−1^ [37]. Hua et al. [39] reported that the ability of *W. anomalus* to the control of *Aspergillus flavus* growth can also be attributed to the secretion of 2-phenylethanol, an antifungal volatile compound, which inhibited spore germination and the expression of the aflatoxin biosynthetic genes of *A. flavus*.

The application of volatile organic compounds does not require the direct contact of antagonistic yeasts with the fruits as the VOCs are absorbed through cell membranes. This leads to a change in the permeability of ions and metabolite diffusion and finally to a loss of the homeostasis of fungal cells [40].

### 3.4. The Thickness and Mechanical Properties of Alginate Films

Some selected mechanical properties of the alginate biofilms were evaluated. The tensile strength (TS) and elongation at break (%E) are parameters describing the mechanical characteristics of the materials [41]. As shown in Table 2, no significant effects on the tensile strength and %E were observed after the addition of killer yeast cells to alginate coatings. The incorporation of killer yeasts into alginate increased the thickness of the films (Table 2). The effect was statistically significant (*p* < 0.05) compared to the control film. The mechanical properties of the alginate biofilms were comparable to those reported by Benavides et al. [41] and Aloui et al. [15].

### 3.5. Survival of Killer Yeasts in Biofilms during Storage

**Figure 1:**
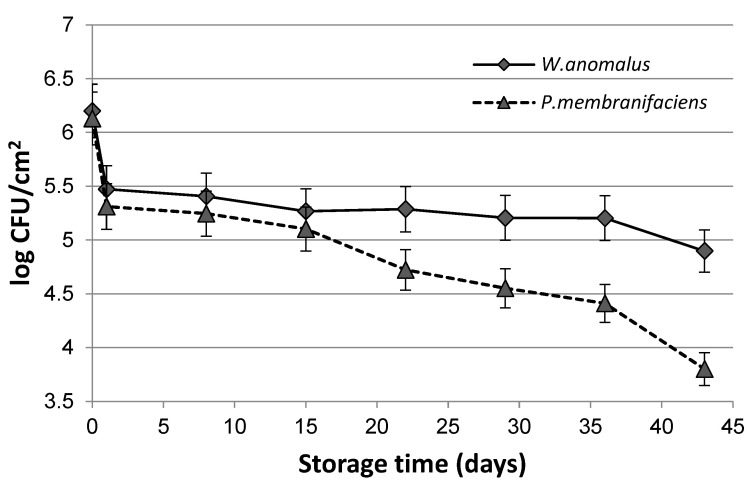
Changes in the population of the *W. anomalus* and *P. membranifaciens* killer yeasts entrapped in sodium alginate during storage (four weeks at 2 °C and 14 days at 22 °C).

The composition of the biofilm should ensure the survival of antagonistic microorganisms and not negatively affect their antifungal activity during storage. The viabilities of *W. anomalus* and *P. membranifaciens* killer yeasts incorporated in the sodium alginate films were evaluated just after the biofilm was dried and every 7 days during storage periods of 28 days at 2 °C, and subsequently, 14 days at 22 °C. The survival of *W. anomalus* cells was higher than *P. membranifaciens*. The population of *W. anomalus* immobilized in the alginate film at the end of the storage remained above 75%, while that of *P. membranifaciens* was at about 60% (Figure 1).

In the previous study, Aloui et al. [15] demonstrated that locust bean gum and sodium alginate films were able to maintain more than 85% of the initial *W. anomalus* BS 91 yeast population after storage for 21 days at 25 °C. The viability of *Debaryomyces hansenii* entrapped in covalently cross-linked arabinoxylans coating was even higher [18]. The arabinoxylan matrix was able to maintain more than 90% viability of the initial inoculum of *D. hansenii* for 20 days at 13 or 25 °C. Settier-Ramírez et al. [20] reported that the viability of *M. pulcherrima* in pectin films was lower than in that of apple pomace films. In addition, Fan et al. [19] reported that the viability of *Cryptococcus laurentii* was better in the presence of a carbon source in the bioactive alginate coating. After 20 days of storage at 20 °C, the population of yeast cells entrapped in the films remained at 49.7%, 81.9%, and 76.6% for the control, 3% carbon source, and 6% carbon source, respectively [18].

The presence of glycerol in the biofilms seemed to be important in maintaining the viability of the antagonistic yeast cells [18]. *W. anomalus* has the ability to utilize glycerol as a carbon source. *P. membranifaciens* assimilates far fewer compounds than *W. anomalus*, and its capability to assimilate glycerol is variable [42]. It is possible that the addition of a carbon source could increase the survival of yeasts in the alginate coating with the tested killer yeasts.

### 3.6. Post-Harvest Control of P. italicum and B. cinerea Growth with Bioactive Coatings on Apples

**Figure 2:**
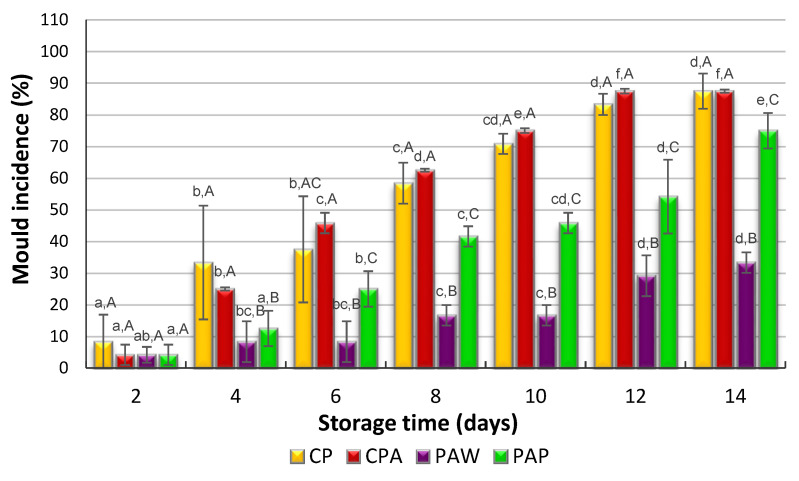
The effect of biofilms with *W. anomalus* or *P. membranifaciens* on the incidence of *P. italicum* on apple fruit. CP: fruit injured and inoculated with *P. italicum*; CPA: fruit injured and inoculated with *P. italicum*, coated with alginate; PAW: fruit injured and inoculated with *P. italicum*, coated with alginate containing *W. anomalus*; PAP: fruit injured and inoculated with *P. italicum*, coated with alginate containing *P. membranifaciens*. a–f: values with different superscript Roman letters (for each storage time) were significantly different (*p* < 0.05); A–C: values with different superscript Roman letters (for each treatment) were significantly different (*p* < 0.05).

**Figure 3:**
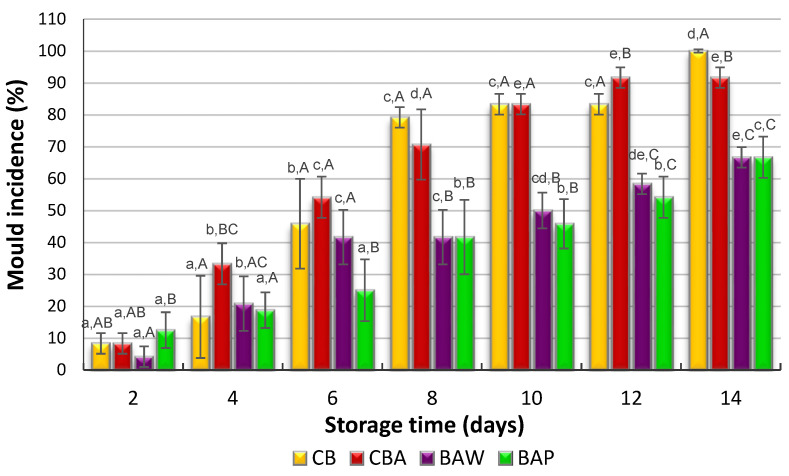
The effect of biofilms with *W. anomalus* or *P. membranifaciens* on the incidence of *B. cinerea* on apple fruit. CB: fruit injured and inoculated with *B. cinerea*; CBA: fruit injured and inoculated with *B. cinerea*, coated with alginate; BAW: fruit injured and inoculated with *B. cinerea*, coated with alginate containing *W. anomalus*; BAP: fruit injured and inoculated with *B. cinerea*, coated with alginate containing *P. membranifaciens.* a–e: values with different superscript Roman letters (for each storage time) were significantly different (*p* < 0.05); A–C: values with different superscript Roman letters (for each treatment) were significantly different (*p* < 0.05).

In the first phase of the experiment, apple fruits inoculated with *P. italicum* or *B. cinerea* were stored for 28 days at 2 ± 0.5 °C and 85–90% RH. However, the control fruits and fruits coated with biofilms containing killer yeasts did not show any signs *of P. italicum* or *B. cinerea* growth after this period of storage. In the second stage of the experiment, the fruits were stored at 22 ± 1 °C and 45% RH for 14 days to simulate the retail handling and market conditions. The ability of treatments to control the post-harvest decay of apples caused by *P. italicum* or *B. cinerea* during storage at 22 °C is presented in Figure 2 and Figure 3.

A maximum infection level (almost 90% or 100%) was observed at the end of the storage (14th day) at 22 °C in the control apples inoculated with *P. italicum* or *B. cinerea*, respectively. There were no major differences in mold incidence between the control fruits and apples coated with sodium alginate without killer yeasts. The alginate coating alone did not protect the fruits against the development of the molds examined. The results obtained for apples inoculated with *P. italicum* (Figure 2) showed that the decay incidence of fruits coated with alginate biofilms containing *W. anomalus* or *P. membranifaciens* was about 35% and 75%, respectively. The killer yeasts *W. anomalus* provided good control of the disease incidence, while *P. membranifaciens* appeared to be significantly less effective in the inhibition of *P. italicum* growth on apples. The weaker ability of this killer strain to inhibit mold growth may be associated with a too low concentration of the killer yeast cells in the bioactive coating.

A bioactive coating with incorporated *W. anomalus* or *P. membranifaciens* killer yeast also reduced the incidence of grey mold during 14 days of the storage of apples inoculated with *B. cinerea* compared with the control fruits (Figure 3), however, the reduction in the fungal decay was from 100% (control) to 67%. The results revealed that *W. anomalus* showed better efficacy against fungal infection caused by *P. italicum* than *B. cinerea*.

The species *W. anomalus* (formerly known as *P. anomala*) has been granted the Qualified Presumption of Safety (QPS) status from the European Food Safety Authority (EFSA) and this allows us to think about its application in enzyme production, and presumably, in food preservation in the future [43,44]. Previously, other reports have shown that some *W. anomalus* killer yeast strains had the ability to control post-harvest disease caused by *Penicillium digitatum* on oranges [15,28] or lemons [23]. Lima et al. [29] assessed the efficiency of the killer yeasts *W. anomalus* (strain 422) as a biocontrol agent against *Colletotrichum gloeosporioides*, causing the anthracnose of papaya and many other tropical fruits. The application of *W. anomalus* on papaya fruits 12 or 24 h before the phytopathogen inoculation resulted in a significant reduction in the lesion size caused by *C. gloeosporioides* (13.75% and 30%, respectively).

Aloui et al. [15] reported that coatings formulated with locust bean gum or sodium alginate enriched with *W. anomalus* BS 91 yeast cells were effective at controlling the incidence of green mold on oranges inoculated with *P. digitatum* until the end of the 13-day storage period, which resulted in a fungal decay reduction in the coated oranges of more than 70% when compared with the control and coatings formulated with pure locust bean gum or pure sodium alginate. This was in agreement with the results of Platania et al. [28], which demonstrated that *W. anomalus* BS 91 showed a strong capability to control *P. digitatum* growth on Tarocco orange fruit until the tenth day of storage. *W. anomalus* strains can produce and release hydrolytic enzymes, which degrade the fungal cell wall, and causes the cell lysis, and may explain their efficacy against some fungal pathogens including *P. digitatum* [28]. The synthesis of exo-β-1,3 glucanases has also been shown to contribute to the mechanism of the antifungal action of the antagonistic yeasts *W. anomalus* (strain K) against *B. cinerea* on apples [3,45] and *W. anomalus* (strain 422) against *C. gloeosporioides* in papaya [29].

Masih et al. [46] found that the *P. membranifaciens* strain FY-101 could be a good biocontrol agent against *B. cinerea*, causing the grey mold disease in a grapevine. The yeast strain exhibited the antifungal activity against *B. cinerea* by secreting cell wall-lytic enzymes such as β-1,3-glucanases and it is one of the possible mechanisms related to the antagonism between *P. membranifaciens* and *B. cinerea* [47]. Santos et al. [35] found that the post-harvest application of the killer strain, *P. membranifaciens* CYC 1106, to wounded apples significantly reduced grey mold lesions. Treatments with concentrations of the killer yeast strain of about 10^6^ cells mL^−1^ resulted in the total inhibition of *B. cinerea* CYC 20010. The similar results obtained in the experiment with the purified killer toxin produced by *P. membranifaciens* CYC 1106 revealed that the killer phenotype should be taken into consideration when the antagonism between fungi and yeasts is under study [35]. It was also found that competition for the available substrates or the secretion of hydrolytic enzymes could be additional synergistic mechanisms by which *P. membranifaciens* CYC 1106 reduced *B. cinerea* CYC 20,010 growth. The smaller diameter of the inhibition zone observed after the application of the purified toxin in comparison with the growing cells of the killer yeast confirmed that additional mechanisms are involved in the antagonistic action of *P. membranifaciens* CYC 1106 [35].

In another study, the development of disease symptoms in *Vitis vinifera* plants inoculated with *B. cinerea* was stopped in the presence of *P. membranifaciens* killer yeast cells or the purified toxin [34]. In this case, the inhibition was stronger when actively growing cells of *P. membranifaciens* were used, which may confirm the occurrence of additional mechanisms of antagonistic activity of *P. membranifaciens* killer yeasts compared to the purified toxin.

### 3.7. Quality Parameters of Apples Uncoated and Coated with Alginate Biofilms

The application of alginate as a carrier to microorganism cells was aimed at limiting the weight loss and prolonging the shelf life of apples. Weight loss is an important factor of the fruit’s post-harvest quality. The weight loss of apples coated with alginate was reduced during storage when compared to the control fruits (Table 3). The incorporation of killer yeast cells into the alginate coatings did not significantly (*p* < 0.05) affect the water loss of apples and these results are in agreement with those previously published by Aloui et al. [15]. A slightly different tendency was observed by Fan et al. [19], who reported a decrease in the weight loss of coated strawberries upon the incorporation of *C. laurentii* into alginate film.

It could be expected that the alginate coating will positively affect the fruit firmness during storage, however, no statistically significant differences were found when compared to the control (Table 3). In the study of Olivas et al. [48], it was found that the alginate coatings extended the shelf life of the cut ‘Gala’ apples. Edible coatings made from alginate, alginate-acetylated monoglyceride-linoleic acid, and alginate-butter-linoleic acid, reduced the weight loss of apple slices during storage. Additionally, the firmness of alginate-coated fruits remained constant throughout the storage period, while in the control group, a large decrease in firmness was recorded. Edible coatings applied as a thin layer can work as a physical barrier against water loss caused by transpiration and respiration processes [45]. The beneficial effect of bioactive coatings on weight loss limitation was also observed for chitosan coatings of papaya [49] and strawberries [50]. In another study, alginate coatings without grapefruit seed extract were applied for the preservation of table grapes [51]. The above-mentioned coatings were also shown to be effective at preventing weight loss and maintaining the firmness of stored grape berries. Similarly, the bioactive coating based on locust bean gum or sodium alginate applied on ‘Valencia’ oranges were efficient in reducing the weight loss compared with the uncoated control fruits. A decrease in weight in the coated oranges was estimated to be 28–33% after 15 days of fruit storage when compared to the uncoated oranges [15].

## 4. Conclusions

Alginate biofilms containing *W. anomalus* killer yeasts could be effective in limiting post-harvest fungal decay losses in organically grown apples. *W. anomalus* killer yeast incorporated in alginate reduced the *P. italicum* incidence from 90% (control) to 35% after 14 days of storage at 22 °C. Alginate coatings with *W. anomalus* or *P. membranifaciens* also limited the incidence of the fungal decay of apples inoculated with *B. cinerea* compared with the control fruits, although the antagonistic capability against *B. cinerea* was lower than that against *P. italicum*. Therefore, the proposed alginate coatings containing killer yeasts seem to be a promising solution by offering the non-chemical, biological control of post-harvest pathogens. Future research will likely focus on determining the optimal biocontrol yeast concentration and composition of the bioactive coatings aiming to improve their antifungal and other functional properties, which will increase the shelf life of apples.

## Figures and Tables

**Table 1:** In vitro antagonistic activity of the killer yeasts against the fungi, expressed as percentages of the fungal colony inhibition.

Yeasts	Fungus	Inhibition by Diffusible Metabolites (%) ^1^	Inhibition by Volatiles Compound (%) ^2^
*W. anomalus*	*P. italicum*	56.2 ± 0.8 ^a^	50.8 ± 3.2 ^a^
	*B. cinerea*	53.3 ± 2.8 ^a^	69.7 ± 5.3 ^b^
*P. membranifaciens*	*P. italicum*	37.8 ± 1.5 ^A^	21.1 ± 4.5 ^A^
	*B. cinerea*	63.9 ± 3.5 ^B^	40.3 ± 4.3 ^B^

^1^ Percentage of mycelial growth inhibition was estimated compared to the control without the killer yeasts after 10 days of incubation; ^2^ Percentage of mycelial growth inhibition estimated compared to the control without the killer yeasts after 5 days of incubation. ^a,b, A,B^: values with different superscript Roman letters in the same column were significantly different (*p* < 0.05).

**Table 2:** The thickness and mechanical properties of the alginate films.

Films	Thickness (mm)	TS (MPa)	%E
A	0.038 ± 0.005 ^a^	40.96 ± 1.44 ^a^	4.90 ± 1.07 ^a^
AWA	0.044 ± 0.002 ^b^	40.18 ± 2.91 ^a^	5.28 ± 1.46 ^a^
APM	0.048 ± 0.005 ^b^	43.49 ± 4.13 ^a^	4.97 ± 1.54 ^a^

A: sodium alginate; AWA: sodium alginate with incorporated *W. anomalus*; APM: sodium alginate with incorporated *P. membranifaciens*; TS: tensile strength; %E: percent elongation at break; ^a, b^: values with different superscript Roman letters in the same column were significantly different (*p* < 0.05).

**Table 3:** The quality parameters of the apples uncoated and coated with alginate biofilms after 28 days storage at 2 °C.

Treatments	Weight Loss (%)	Firmness (N)	Extract (%)	Titratable Acidity ^1^ (g L^−1^)
CP	1.75 ± 0.37 ^a^	53.4 ± 4.3 ^a^	13.3 ± 0.3 ^a^	5.34 ± 0.10 ^a^
CPA	0.93 ± 0.12 ^bc^	57.2 ± 5.7 ^a^	14.2 ± 0.4 ^b^	4.96 ± 0.26 ^ab^
PAW	0.82 ± 0.15 ^b^	55.6 ± 3.2 ^a^	13.7 ± 0.5 ^ab^	5.63 ± 0.11 ^c^
PAP	1.17 ± 0.20 ^c^	56.1 ± 2.7 ^a^	13.9 ± 0.2 ^b^	4.77 ± 0.12 ^b^
CB	1.80 ± 0.28 ^A^	55.5 ± 3.3 ^A^	13.6 ± 0.3 ^A^	5.64 ± 0.18 ^A^
CBA	0.98 ± 0.15 ^B^	57.0 ± 6.1 ^A^	14.0 ± 0.2 ^B^	4.79 ± 0.38 ^B^
BAW	1.10 ± 0.18 ^B^	56.1 ± 3.1 ^A^	13.9 ± 0.3 ^AB^	5.55 ± 0.04 ^A^
BAP	1.09 ± 0.15 ^B^	55.1 ± 5.4 ^A^	13.6 ± 0.2 ^AB^	5.75 ± 0.08 ^A^

CP: fruit inoculated with *P. italicum*; CPA: fruit inoculated with *P. italicum*, coated with alginate; PAW: fruit inoculated with *P. italicum*, coated with alginate containing *W. anomalus*; PAP: fruit inoculated with *P. italicum*, coated with alginate containing *P. membranifaciens*; CB: fruit inoculated with *B. cinerea*; CBA: fruit inoculated with *B. cinerea*, coated with alginate; BAW: fruit inoculated with *B. cinerea*, coated with alginate containing *W. anomalus*; BAP: fruit inoculated with *B. cinerea*, coated with alginate containing *P. membranifaciens*; ^1^–expressed as g⋅L^−1^ of malic acid; a–c, A,B: values with different superscript roman letters in the same column are significantly different (*p* < 0.05).

## Data Availability

The data presented in this study are available on request from the corresponding author.
